# An Effective Method for Lung Cancer Diagnosis from CT Scan Using Deep Learning-Based Support Vector Network

**DOI:** 10.3390/cancers14215457

**Published:** 2022-11-06

**Authors:** Imran Shafi, Sadia Din, Asim Khan, Isabel De La Torre Díez, Ramón del Jesús Palí Casanova, Kilian Tutusaus Pifarre, Imran Ashraf

**Affiliations:** 1 College of Electrical and Mechanical Engineering, National University of Sciences and Technology, Islamabad 44000, Pakistan; 2 Sadia Din Texas A&M University at Qatar, Education City, Al Rayyan 23874, Qatar; 3 Department of Computing, Abasyn University Islamabad Campus, Islamabad 44000, Pakistan; 4 Department of Signal Theory and Communications and Telematic Engineering, University of Valladolid, Paseo de Belén 15, 47011 Valladolid, Spain; 5 Research Center for Foods, Nutritional Biochemistry and Health, Universidad Internacional Iberoamericana, Campeche 24560, Mexico; 6 Research Center for Foods, Nutritional Biochemistry and Health, Universidad Internacional Iberoamericana, Arecibo, PR 00613, USA; 7 Inovation Projects Department, Universidad Europea del Atlántico, Isabel Torres 21, 39011 Santander, Spain; 8 Research Center for Foods, Nutritional Biochemistry and Health, Universidade Internacional do Cuanza, Cuito EN 250, Angola; 9 Fundación Universitaria Internacional de Colombia, Calle 39A #19-18, Bogotá 111311, Colombia; 10 Department of Information and Communication Engineering, Yeungnam University, Gyeongsan 38541, Korea

**Keywords:** lung cancer detection, capsule neural network, wide network, computed tomography

## Abstract

**Simple Summary:**

This study provides an efficient method for lung cancer diagnosis from computed tomography images and employs deep learning-supported support vector machine. Experimental results indicates that the proposed approach yields a 94% accuracy and performs better than existing models.

**Abstract:**

The diagnosis of early-stage lung cancer is challenging due to its asymptomatic nature, especially given the repeated radiation exposure and high cost of computed tomography(CT). Examining the lung CT images to detect pulmonary nodules, especially the cell lung cancer lesions, is also tedious and prone to errors even by a specialist. This study proposes a cancer diagnostic model based on a deep learning-enabled support vector machine (SVM). The proposed computer-aided design (CAD) model identifies the physiological and pathological changes in the soft tissues of the cross-section in lung cancer lesions. The model is first trained to recognize lung cancer by measuring and comparing the selected profile values in CT images obtained from patients and control patients at their diagnosis. Then, the model is tested and validated using the CT scans of both patients and control patients that are not shown in the training phase. The study investigates 888 annotated CT scans from the publicly available LIDC/IDRI database. The proposed deep learning-assisted SVM-based model yields 94% accuracy for pulmonary nodule detection representing early-stage lung cancer. It is found superior to other existing methods including complex deep learning, simple machine learning, and the hybrid techniques used on lung CT images for nodule detection. Experimental results demonstrate that the proposed approach can greatly assist radiologists in detecting early lung cancer and facilitating the timely management of patients.

## 1. Introduction

Lungs are air-filled organs within the thoracic cavity (chest) and constitute the main part of the human respiratory system. The cells within the lung may undergo cancerous change (malignancy) giving rise to lung cancer, which is the leading cause of cancer-related deaths, accounting for around 27% [1]. Mutation, unregulated tissue growth, refers to the occurrence of permanent changes in deoxyribonucleic acid (DNA) sequences. Mutation can be due to external factors or inherited genetic abnormalities. The most common external factors are chemicals in tobacco smoke but numerous other carcinogens exist. The damaged tissue is usually replaced by new tissue but in the presence of a malignant mutation, the new tissue growth is unregulated and leads to cancerous cells. Lung nodules are abnormal growths within the lung that can either be benign or malignant and most are benign, but some can be a sign of early cancer (suspicious nodules). Recent studies estimate the 5-year survival rate of lung cancer to be only about 19% [2]. Lung cancer survival is markedly increased by early diagnosis (i.e., diagnosis of early-stage lung cancer). Benign nodules are noncancerous and do not spread to other parts of the body. According to World Health Organization (WHO), the rate of cancer-related deaths is expected to increase to 45% by 2030 [3].

Morphologically, the malignant cells are characterized by a nucleus with irregular shape and size [4]. Commonly, morphological features of the CT images are used to analyze benign and malignant nodules. The straightforward approach heavily relies on high-level and experienced radiologists for judging benign and malignant tumors. Due to the proportionality in features, making a comprehensive judgment becomes difficult. Another approach to overcome subjectivity is to train classifiers to automatically classify benign and malignant nodules based on morphological features. Different methods can be employed including using single classifiers and multiple features with classifiers [5]. The early detection of nodules provides a better chance for a patient’s survival. Therefore, identifying the potential malign lung nodules becomes essential in diagnosing lung cancer. To detect lung cancer, the characteristic of the benign nodule is compared with malignant ones [6]. There is a considerable overlap of features of benign and malignant nodules, and morphologic features need to be evaluated carefully for effective nodule assessment. Furthermore, morphological assessment is essential for early diagnosis [7].

The timely detection of suspicious nodules can be achieved by performing computed tomography (CT) scans. The scanned images of the body’s internal structure demonstrate details by providing front, bottom, and top views, respectively. More information about bones, soft tissues, and blood vessels is available when compared with plain X-rays. However, it is difficult to detect early-stage lung cancer using chest CT scans, due to the similarity of lung nodules to surrounding structures (e.g., blood vessels). Therefore, there is a pressing need to design a computer-aided design (CAD) system to detect suspicious nodules. Deep learning is a promising tool for classifying benign and malignant cancer nodules by potentially reducing the number of scans required to achieve a benign or malignant diagnosis. Deep learning techniques show promising results to detect the benign and malignant modules [8]. The current, state-of-the-art, CAD system uses deep learning models to classify lung nodules, to detect whether it is suspicious or not. Such systems are also used to classify the type of nodule as if it is suspicious or benign.

**Figure 1:**
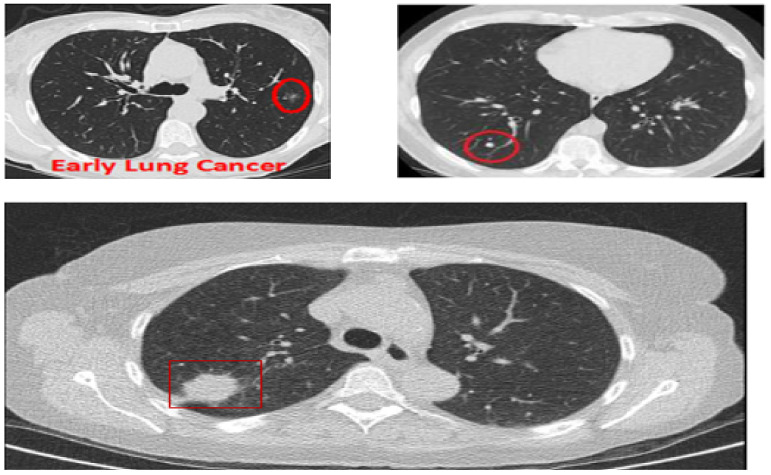
CT scans based on maximum intensity projection.

Although CAD systems demonstrate significantly high efficiency in lung nodule detection, the number of studies considering the routine workflow of radiologists is relatively low. Clinically, radiologists analyze the maximum projection intensity (MIP) images to locate the nodular candidates for further examination. MIP allows projecting 3-D voxels with maximum intensity to the plane of projection, thus enhancing nodule visualization [9]. MIP images are not threshold dependent and allow for preserving attenuation information [10], which helps convolutional neural networks (CNN) automatically detect lung nodules. In Figure 1, three CT images are shown: The top two show lungs containing early-stage cancer; the first being malignant and the second being benign. It is difficult to differentiate between vessel and cancer nodules during this stage. The last image is of cancer in its late stage, with a large nodule size making it easier to detect, but survival rates are low.

This paper proposes an effective method to classify benign and malignant nodules from CT images. To take the advantage of rich information without sacrificing efficiency, MIP is employed to classify lung cancer nodules as benign or malignant. This study aims to develop an effective method to classify benign and malignant lung cancer nodules using CT scans based on MIP. The performance is analyzed in comparison to several hybrid models, which are specifically designed for lung cancer detection.

## 2. Materials and Methods

**Figure 2:**
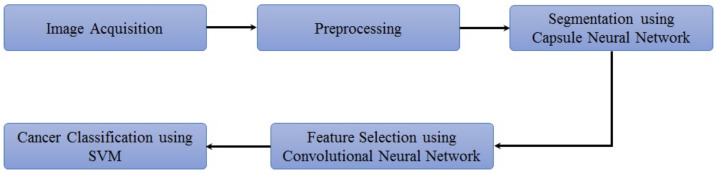
Flow of the proposed approach.

**Figure 3:**
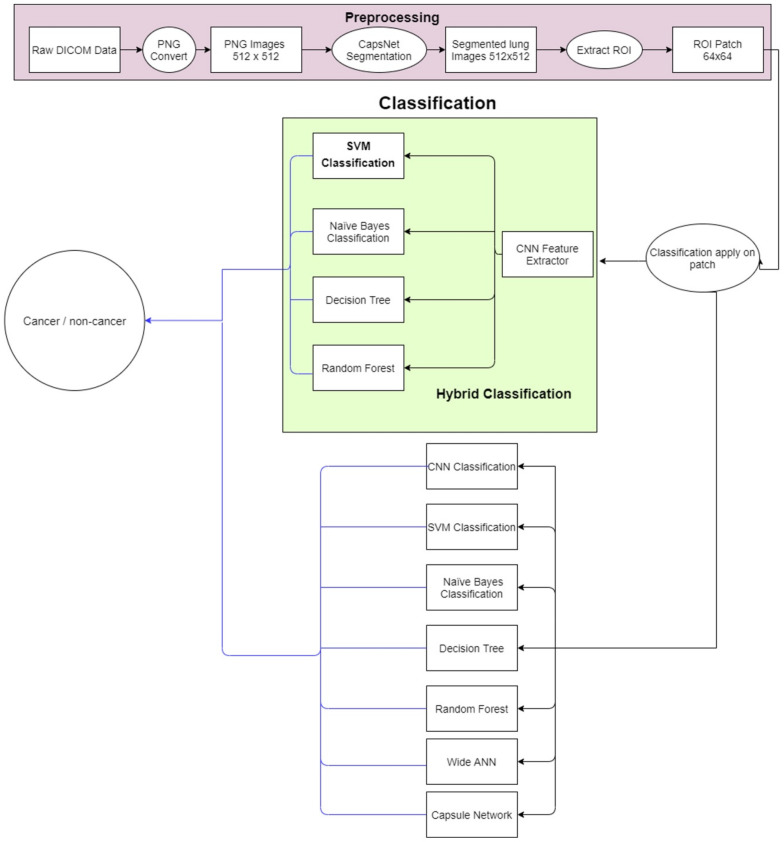
Flow diagram of the proposed system.

The proposed methodology is depicted in Figure 2. The lung nodule image dataset is obtained and preprocessed initially. Then, lung segmentation is conducted by capsule network [11]. Capsule segmentations demonstrate state-of-the-art results as compared to other techniques such as U-net [12] and image processing techniques. After lung segmentation, the region of interest (ROI) is selected using the information provided in the LUNA16 dataset. Then on that ROI, the proposed hybrid technique is applied for the classification of cancerous and non-cancerous cells. The architecture of the proposed approach is shown in Figure 3. The acquired images are preprocessed and segmented to extract the region of interest (ROI). Next, a hybrid technique is applied for the classification of cancerous and non-cancerous cells. Acronyms used in this study can be found in Table S1.

### 2.1. Image Preprocessing and Segmentation

#### 2.1.1. Dataset

The LUNA16 dataset [13] is used, which contains CT scanned images in DICOM format. The dataset is created from a publicly available LIDC/IDRI database, and a slice thickness of greater than 2.5 mm is excluded. The dataset contains a total of 888 samples, where the CT scanned images have a 512 × 512 × Z resolution (Z being the depth of DICOM Format CT scan, which varied for each CT sample, ranging from 100 to 400). Four experienced radiologists marked the annotation of nodules and non-nodule. The reference standard nodule is accepted, if 3 out of the 4 radiologists marked it as a nodule and the size of that nodule is recorded to be greater than 3 mm. The location of each nodule in the lung is given with a label to help identify whether it is cancerous. The size of each nodule is also mentioned, with 1186 annotations being available. The total size of the dataset is 128 GB.

#### 2.1.2. Data Preprocessing and Segmentation

A deep learning-based capsule network CapsNet [11] is utilized to maintain the objects’ position and properties. Capsule-based segmentations demonstrate state-of-the-art results compared to other techniques such as U-net [12]. A 3-layer block capsule is used. The input of the 512 × 512 image is given to the input layer of the capsule. Figure S2 shows the capsule network model used for lung segmentation.

After applying SegCaps on lung images, a segmented lung image is obtained. From the segmented lungs, a 64 × 64 lung patch is extracted. The extracted patch of 64 × 64 pixel is then used with deep learning-enabled support vector machine (SVM) methodologies for detecting cancerous nodules in the patch. A total of 2400 patch images (1200 cancerous and 1200 non-cancerous) existed, of which 200 samples (100 cancerous and 100 non-cancerous) are used for training.

### 2.2. Nodule Classification Using Deep Learning Enabled Support Vector Machine

The features extracted by the neural network are classified on the SVM. In SVM, the best hyperplane (decision boundary) is identified in the *N* number of features for the best classification, hence detecting the cancer nodule type. SVM requires less memory to run and is relatively memory efficient than other machine learning algorithms. SVM is more accurate if the number of dimensions is greater than the number of samples. It also works well when clear margin space exists in classes and is robust on outliers. However, on a large dataset, SVM does not work well and is not suitable. The application of the kernel function becomes difficult when the dimension for classification is increased.

In deep learning (DL) enabled SVM, deep-learning techniques are applied to detect lung cancer. Firstly, the convolution neural network is trained with backpropagation on lung patches. After training, this network is splatted from the flattened layer. Hence, the convolution layers block and fully connected layers block become separated. The convolution layer block is then ready to extract features. After flattening, the features are given to the support vector machine for classification. In a pure machine learning model, handcrafted features are needed for classification, while in a deep learning model, features can be extracted themselves. Therefore, in a hybrid approach, feature selection is carried out by convolution layers, and classification is conducted by SVM.

**Figure 4:**
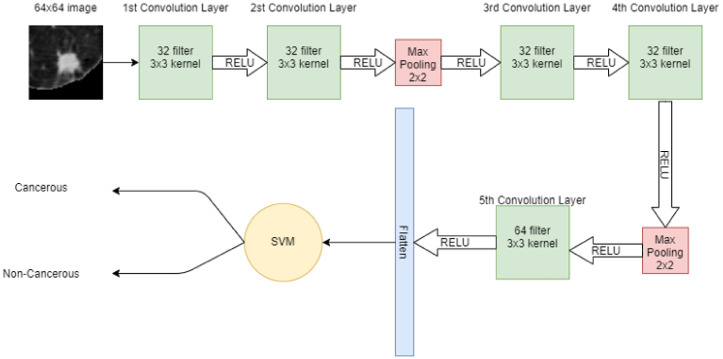
Architecture of proposed hybrid model (CNN + SVM).

A 64 × 64 image is given to the convolutional layer. The network consists of 5 convolutional layers in total. The first two convolutional layers have 32 filters with 3 × 3 kernel. The next convolutional layer has 2 × 2 max pooling, then more two-convolution layers are placed with 32 filters and 3 × 3 kernel. Again, the max-pooling 2 × 2 is applied and then in the 5th convolutional layer, 64 filters with 3 × 3 kernel are present. Finally, the output of the 5th convolutional layer is flattened in a one-dimensional array. In that 1D vector, features are extracted. These features are then input to the machine learning model SVM for classification. Figure 4 shows the hybrid model, in which CNN extracts the features from the image and SVM is used for classification.

### 2.3. Base for the Lung Cancer Detection Model: Algorithm of Support Vector Machine

For binary class problems, many possible hyperplanes distinguish two-class problems. However, in SVM, the best hyperplane among all has been identified based on the maximum margin. If there is a *W* weight vector and an *X* feature, then the mathematical equation of the hyperplane is written as

(1) $$W^{T}X+b=0$$

SVM classifies the data point in 1 class if $W^{T}X+b\geq 1$ and another class if the data point is $W^{T}X+b<1$. Thus, in general, the hypothesis function for the classification of cancerous and non-cancerous using SVM is

(2) $$h\left(x_{i}\right)=\left\{\begin{array}{cc} cancer & if\;w.x+b\geq 0\\ non-cancer & if\;w.x+b<0 \end{array}\right.$$

Another important thing in SVM is the selection of kernel. Kernel helps SVM to separate the non-separable data by adding a new feature and drawing the hyperplane. Data are then separated on high dimensionality and a hyperplane can be drawn. This study uses a radial basis function (RBF) kernel to compute the closeness and similarity between two points. If $X_{1}$ and $X_{2}$ are two points, then RBF can mathematically be represented as

(3) $$K\left(X_{1},X_{2}\right)=exp\left(\frac{-\left|\right|X_{1}-X_{2}{\left|\right|}^{2}}{2\sigma^{2}}\right)$$

where $\left|\right|X_{1}-X_{2}\left|\right|$ is the Euclidean distance between the given points and σ is the variance hyperparameter.

If Euclidean distance is valued as 0, it concludes that $X_{1}=X_{2}$, and hence both points are the same. When the kernel value is less than 1 and close to 0, the points are dissimilar. Therefore, it is necessary to find the appropriate value of σ to determine which points to consider. The distance can be considered as the dissimilarity between points $X_{1}$ and $X_{2}$, because if the distance decreases, the point is much similar and if the distance increases, and then $X_{1}$ and $X_{2}$ are considered dissimilar. The value of σ can be considered as the base if σ=1 then $\sigma^{2}=1$ and RBF equation becomes

(4) $$K\left(X_{1},X_{2}\right)=exp\left(\frac{-\left|\right|X_{1}-X_{2}{\left|\right|}^{2}}{2}\right)$$

The graph for Equation (4) is for σ=1; if the distance between two points is less than 4, these points are considered similar and if the distance is greater than 4, then these points are considered dissimilar. If a small σ is assumed, e.g., σ=0.1 then $\sigma^{2}=0.01$ and RBF equation become

(5) $$K\left(X_{1},X_{2}\right)=exp\left(\frac{-\left|\right|X_{1}-X_{2}{\left|\right|}^{2}}{0.01}\right)$$

Consequently, the width of the similarity region should become small. The points are only considered similar if their Euclidean distance is found to be less than or equal to 0.2. However, if the distance is large, these points are considered dissimilar. Therefore, it can be concluded that if a large σ value is considered, then similarity has existed and if a small σ value is considered, less distance point is also considered as dissimilar.

### 2.4. Implemented Machine Learning and Deep Learning Models

Experiments are also performed on other techniques for the classification of segmented patches. This study implements several machine learning (ML), DL, and hybrid models, which are DL-enabled ML models. Features extracted from CNN are fed as input to the Naïve Bayes (NB) algorithm, decision tree (DT), and the ensemble learning algorithm random forest (RF), to classify cancerous and non-cancerous nodules. Hybrid models are formed, respectively. In this method, a combination of deep learning and machine learning techniques is used. First, deep learning is used for feature selection and then these features are classified into machine learning classifiers. Necessary architecture and implementation details are provided here.

#### 2.4.1. Capsule Network

To overcome the limitation of CNN, capsule network (CapsNet) came in a capsule network instead of the max pooling capsule layer, which is used to detect the features [14]. In the capsule layer, a single capsule (the set of neurons) acts to identify size, position, and hue. The output of the capsule consists of the length of the vector that determines the probability of features present in the input, and the orientation of the vector is used for the qualification of the capsule property. Capsules are independent and therefore, when multiple capsules are brought to an agreement, the probability of result detection became higher.

The capsule network consists of 2 parts: The encoder and the decoder. The encoder part consists of the convolutional layer, the primary capsule, and the nodule capsule. The convolutional layer is used to detect the basic features. A 64 × 64 image is given to the convolutional layer, which has a 64 × 9 × 9 filter with a stride of 1 that leads to a 56 × 56 feature map. The second layer is the primary capsule layer. The output of the convolutional layer is given to the primary capsule, and the primary capsule makes the combination of input features. It consists of 8 dimensions and has 32 component capsules with 24 × 24 feature maps. The nodule capsule is the last layer in the encoder, containing 2 capsules: one capsule contains cancerous nodule features while the other contains non-cancerous nodule features. The dimension of these two capsules is 16. On the other hand, the capsule decoder has 3 fully connected layers that take input from the nodule capsule and reconstruct the image. In the capsule decoder, 1st layer contains 512 neurons, 2nd layer contains 1024 neurons and the 3rd layer contains 4096 neurons. At the 3rd neuron, the image size that is recreated is 64 × 64.

#### 2.4.2. Convolutional Neural Network

**Figure 5:**
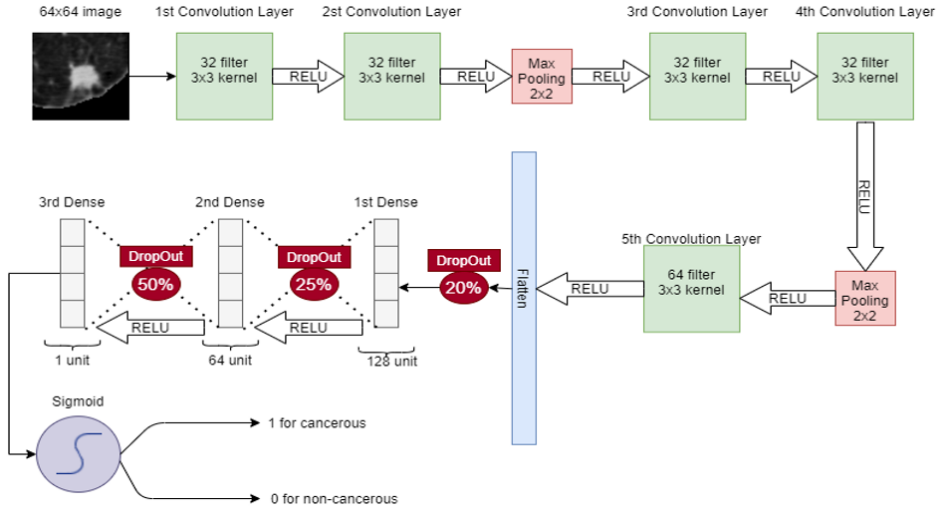
Proposed CNN for classification of nodules (Cancerous-1, Non-Cancerous-0).

In CNN, a 64 × 64 image is given to the convolutional layer. There are 5 convolutional layers and 2 max-pooling layers, stacked in the network. The first convolutional layer has 32 filters with 3 × 3 kernel and a stride of 1. The second convolutional layer also has 32 filters with 3 × 3 kernel and a stride of 1. After these convolutional layers, a 2 × 2 max pooling is applied for feature selection. Later, two convolution layers are placed with 32 filters and 3 × 3 kernel and again max-pooling 2 × 2 is applied. Then, in the 5th convolutional layer, the 64 filter exists with 3 × 3 kernel. After that, the output of the 5th convolutional layer is flattened in a 1-dimensional vector. Three dense connected layers exist after flattening to avoid overfitting. Dropouts of 20%, 25%, and 50% are used before each dense layer, respectively. After each convolutional layer, sigmoid activation functions are used. The proposed CNN for classification is shown in Figure 5.

#### 2.4.3. Wide Neural Network

**Figure 6:**
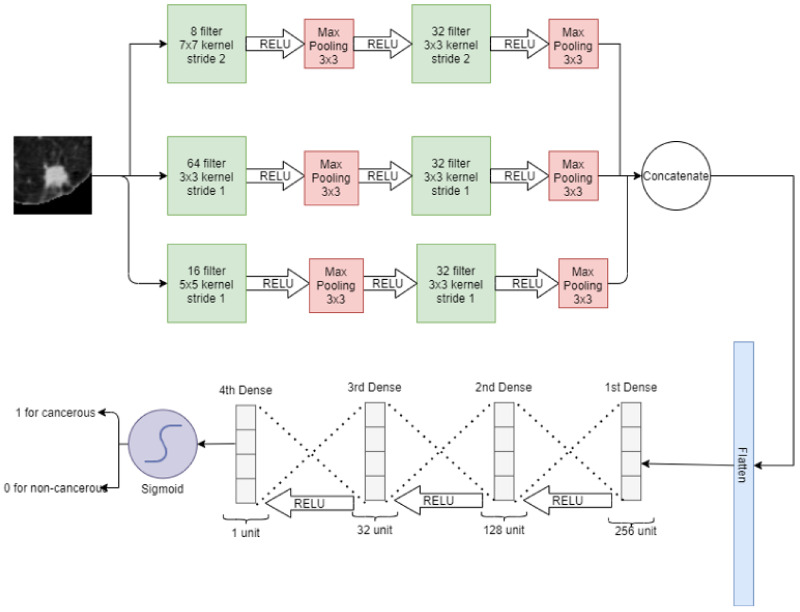
Wide neural net for classification of lung nodules.

In a wide neural network, three convolutional layers are placed parallel to take the input image of a nodule of 64 × 64. For these convolution layers, 8, 64, and 16 filters with kernel size 7 × 7, 3 × 3, and 5 × 5 are used, respectively. After that, a 3 × 3 max pooling is applied at each convolution layer. Again, after each pooling layer, a convolution layer is applied with 32 filters with 3 × 3 kernel size. Then 3 × 3 max pooling is applied and the output of each max pooling is concatenated and flattened in a 1D vector array. Later, 4 fully connected layers are stacked: the first layer has 256 neurons, the second layer has 128 neurons, the third layer has 32 neurons and the fourth layer has only 1 neuron. The sigmoid activation function is then applied for classification. In a wide neural network, convolution layers have different filter sizes to pick the best filter for feature selection. On the first convolution layer, there is a stride of 2 whereas on the rest of the convolutions, there is a stride of 1. The wide neural network for the classification of lung nodules is depicted in Figure 6.

#### 2.4.4. Deep Leaning Enabled Naïve Bayes

**Figure 7:**
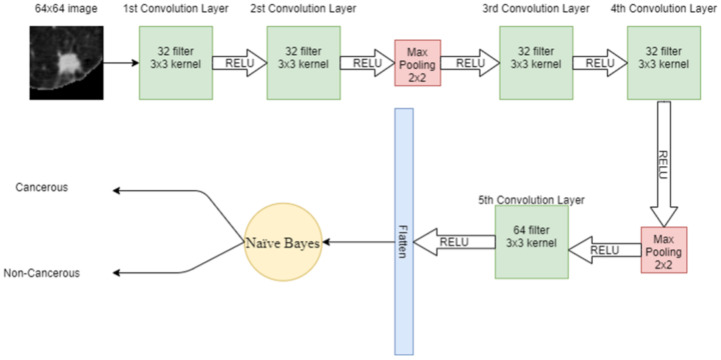
Proposed hybrid model using CNN with NB.

Features extracted through CNN are given as input to the NB algorithm to classify cancerous and non-cancerous nodules. NB is the supervised machine learning algorithm that assumes no correlation between input data. It also performs very well on a small amount of data. First, the features are denoted with $X=(x_{1},x_{2},x_{3},\ldots ,x_{n})$ and class variables by $C_{k}$ Bayes theorem is written as

(6) $$P\left(C_{k}|X\right)=\frac{\left(X|C_{K}\right)P\left(C_{k}\right)}{P(X)},\;fork=1,2,\ldots ,K$$

where $P(C_{k}|X)$ is the posterior probability, $P(C_{k})$ is the prior class probability, $P(X/X_{k})$ is the likelihood and P(X) is the prior probability of the predictor. Figure 7 shows the proposed hybrid model combining CNN with NB.

#### 2.4.5. Deep Leaning Enabled Decision Tree

**Figure 8:**
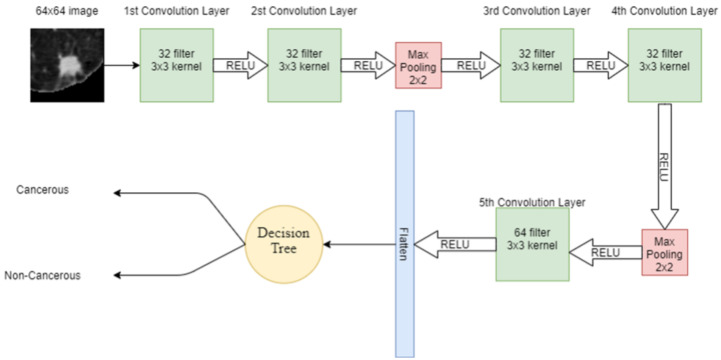
Proposed hybrid model combining CNN and DT.

DT is another supervised machine learning algorithm used in this study. Features extracted from the input lung image through CNN are given to the DT for classification. DT has a tree-like structure, in which, on the root node, there is the most important splitting feature. Internal node attributes are tested and split on the base of the test result, e.g., the data point satisfying one condition. The data are split on one side while the data point satisfying other conditions are split into other sides. The leaf node in the tree represents the available classes in the dataset. In DT, attribute selection is the most important feature, which divides the data in such a way that it results in the output class. The attribute that has the best score is selected as the splitting attribute. Information gain is one of the attribute selection measurements, which gives the split attribute. The hybrid model combining CNN and DT is shown in Figure 8.

(7) $$info\left(d\right)=\sum p_{i}log_{2}\left(p_{i}\right)$$

(8) $$info_{a}\left(D\right)=\sum_{j=1}^{v}\frac{|D_{j}|}{\left|D\right|}\times info\left(D_{j}\right)$$

where $P_{i}$ denotes the probability of attribute into dataset *D*, which belongs to class $C_{i}$. The mean value of the class/category of a data point in *D* is known as info(D) and it is also known as the Entropy of dataset *D*. In most cases, the information is encoded in bits, so a log with a base 2 function is used for that. Information gain can also be calculated as

(9) $$Gain\left(A\right)=info\left(D\right)-info_{a}\left(D\right)$$

#### 2.4.6. Deep Leaning Enabled Random Forest

**Figure 9:**
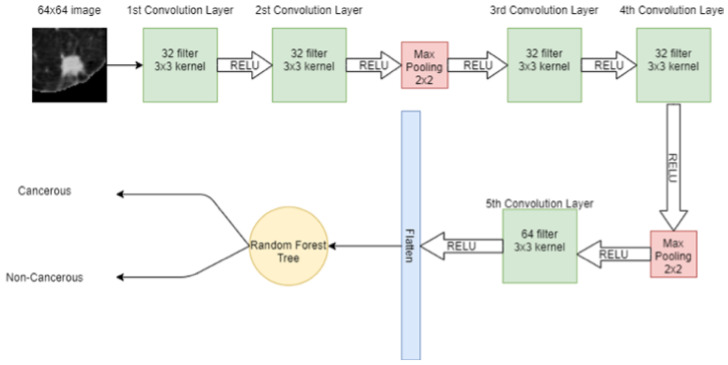
Hybrid model CNN + RF.

RF is an ensemble learning algorithm where many weak classifiers are combined to obtain a strong classifier. In RF, many DTs are trained on the data and each DT acts as one predictor to produce the output class. The class with a majority vote then becomes the final prediction of RF. The hybrid model combining CNN and RF is shown in Figure 9.

### 2.5. Environment

The system utilized for model creation is a NVIDIA graphical processing unit (GPU) with a CUDA compute capability 8.0 on a Windows 10 operating system. The software on which the model is implemented includes Python 3.9, and Keras on top of Anaconda.

### 2.6. Performance Metrics

For performance measurement, multiple metrics are utilized, including accuracy, precision, recall, and F1-score.

The accuracy of the model describes how well the model performs across classes. Accuracy is formulated as shown below in Equation (10).

(10) $$Accuracy=\frac{\mathrm{Number}\;\mathrm{of}\;\mathrm{truly}\;\mathrm{classified}\;\mathrm{samples}}{\mathrm{Total}\;\mathrm{samples}}$$

Precision is the measure of the models’ capability to identify true positives, and it is calculated as shown in Equation (11).

(11) $$Precision=\frac{\mathrm{True}\;\mathrm{Positive}}{\mathrm{True}\;\mathrm{Postive}\;+\;\mathrm{False}\;\mathrm{Positive}}$$

The recall is the ratio between the true positive prediction values and the sum of predicted true positive and false negative values. It is calculated as represented in Equation (12).

(12) $$Recall=\frac{\mathrm{True}\;\mathrm{Positive}}{\mathrm{True}\;\mathrm{Postive}\;+\;\mathrm{False}\;\mathrm{Positive}}$$

F1 score is the overall model accuracy that balances precision and recall in a positive class. It is calculated as represented in Equation (13).

(13) $$F1\;score=2\times \frac{Precision\times Recall}{Precision+Recall}$$

## 3. Results

The LUNA16 dataset is divided into training, validation, and test sets with 710 images in the training set, 89 images in the validation set, and the remaining 89 in the test set. No external validation is applied in this work.

The reported accuracies are based on the comparison of the standalone classification models with the proposed SVM + CNN model for lung nodule classification on the LUNA16 dataset. It was found that the proposed model outperforms standalone models. To further investigate the efficacy of the proposed hybrid model, it is compared with state-of-the-art approaches and DL models.

### 3.1. Experimental Results of All Models

Experiments with the LUNA16 dataset are performed with all the models previously described. In addition, standalone models are also implemented including SVM, DT, RF, and NB to analyze their performance in comparison to the proposed model. Table 1 shows the results regarding the accuracy, precision, recall, and F1 scores. These are the most commonly used performance evaluation metrics and are convenient when compared with existing models.

Results demonstrate that standalone machine learning models demonstrate poor results as compared to hybrid models. In the existing literature, ensemble models are reported to demonstrate a superior performance than single models [15,16,17]. Similarly, current results demonstrate that when DL-enabled machine learning models are used, the classification accuracy is significantly higher. For example, the 72% accuracy of DT is increased to 89% when joined with CNN. The same is true for RF and NB. The highest accuracy for standalone models is RF, i.e., 81%, which is increased to 90% when it is used with CNN. Hybrid models outperform standalone models by a substantial margin. On average, the performance of hybrid is 23% higher than individual models. For hybrid models, the proposed CNN+SVM model demonstartes the highest score of 94%, followed by CNN with a 92% accuracy score. Similarly, the performance of CNN, CapsNet, and the wide artificial neural network (ANN) is poor as compared to the proposed hybrid model. CNN shows better results as compared to the CapsNet and wide ANN.

**Figure 10:**
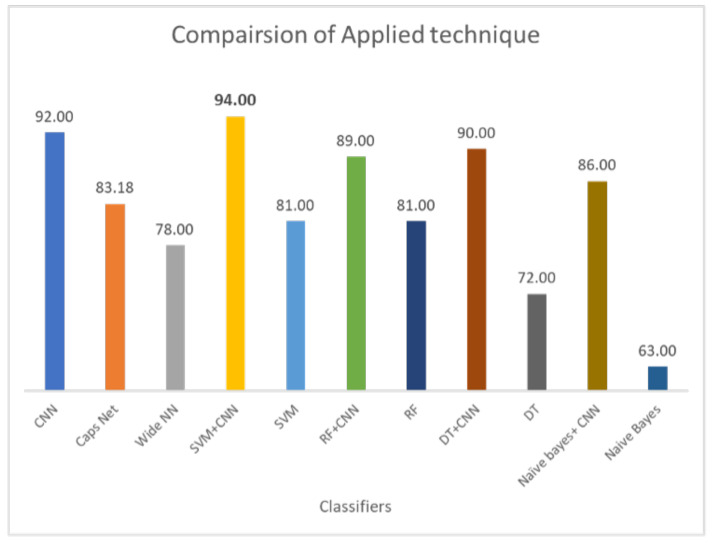
Comparison of all applied techniques on LUNA16 dataset.

Classification of lung nodules is conducted using different methodologies. These include purely machine learning-based methods such as SVM, NB, DT, and RF. DL-based methods such as CNN and using hybrid approaches involve both DL and ML-based approaches such as the deep learning-enabled SVM, NB, DT, and RF. The comparison of applied techniques with the proposed model is shown in Figure 10. The results demonstrate that the CNN selects the important features by max-pooling layer and classifies those features using a fully connected layer. However, the max-pooling layer is inefficient in preserving spatial information and, therefore, loses the information. It works as a messenger between two layers, transferring relevant information, and dropping irrelevant information (from lower to high layers). CNN needs a large amount of training data to train the network; however, a limited amount of data is provided for lung cancer detection. A fully connected layer is capable of classification, but it requires a lot of computation power and input data to train, therefore higher chances of overfitting the data are predicted. The result of CNN is highly dependent on the quality and size of input data; with good quality and large-sized training data, it can surpass humans. However, CNN is not robust on glare and noisy data.

On the other hand, the capsule network methodology does not perform well compared to hybrid models and the CNN model. It also takes comparatively more time for training. Weight initialization is crucial for the capsule network; if the normalization value of weight is set too high it saturates the squashing function, resulting in uniform predictions. However, if the normalization value is set too low, issues arise in later layers due to the squashing of the normalization value. Dynamic routing in the capsule networks is also unstable. Moreover, wide networks have lower accuracy of 78% than other methodologies. The accuracy improvement experiment can be conducted by giving a wide network of 3D input for classification. In a wide network, features can be learned at various levels of abstraction. Multiple layers are good for generalization because they can learn all intermediate features between input data and high-level classification. However, if the wide network becomes too wide, it requires more resources and time to train.

### 3.2. Performance Comparison of Proposed Model with Existing CAD Studies

For performance comparison, several existing state-of-the-art models are selected that conduct experiments using the same LIDC-IDRI or LUNA16 dataset. In addition, some private datasets with fewer samples are also used in these CAD systems. Results are presented regarding the reported accuracy and the number of scans used to obtain that accuracy. Often, a higher number of scans are associated with a higher accuracy score. Table 2 shows the results of all models in comparison to the result of the proposed model. Among all these CAD Systems, the proposed hybrid model shows the best performance on the LUNA16 dataset.

**Figure 11:**
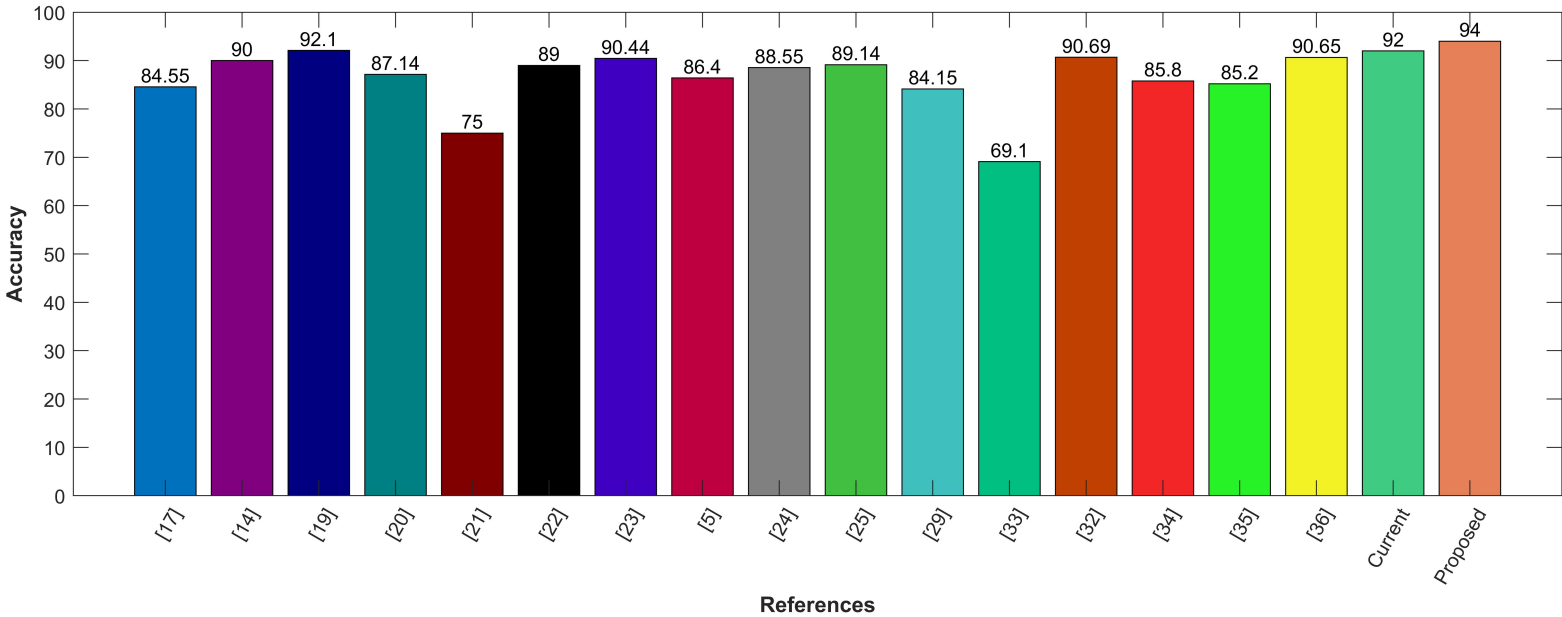
Comparison with other existing CAD systems.

Following is the graphical comparison of different CAD systems that used LIDC/IDRI or LUNA16 datasets. The literature suggests that state-of-the-art AI systems for lung nodule detection and characterization come close to the performance levels of experienced radiologists. The comparison demonstrates that the proposed model utilizing CNN for feature selection and SVM for classification outperforms state-of-the-art methods with the highest accuracy of 94%. The proposed hybrid approach provides feature selection using convolution layers and classification by leveraging SVM. It provides precise information regarding the lung nodules with good sensitivity of 95%. The proposed model aims to reduce the risk of medical errors and provide the confidence to make follow-up decisions in a well-informed way. To demonstrate the efficacy of the presented hybrid model, it is compared with other existing CAD systems, including Google Net, Deep CNN, MC-CNN, and others as shown in Figure 11.

## 4. Discussions

The results demonstrate that the CNN selects the essential features by the max polling layer and classifies those features using a fully connected layer. However, the max polling layer is inefficient in preserving spatial information. It works as a messenger between two layers, transferring relevant information and dropping irrelevant information (from lower to high layers). CNN needs lots of training data to train the network; however, a limited amount of data is provided for lung cancer detection. A fully connected layer is capable of classification, but it requires a lot of computation power and input data to train, therefore, a lot of chances of overfitting the data are predicted. The result of CNN is highly dependent on the quality and size of input data; with good quality and large training data, it can surpass humans. However, CNN is not robust on glare and noise data. This study performs experiments using the LUNA16 lung cancer dataset only and further experiments are intended in the future to analyze the performance of the proposed approach. Moreover, using transfer learning is under consideration to reduce the training time.

Lung nodule detection techniques found in the literature are mostly based on digital image processing (DIP), ML, and DL due to advancements in data acquisition, storage, and processing equipment. ML and DL studies provide different levels of sensitivity and specificity for lung cancer detection, as stated in [31]. Zheng et al. propose a CNN based on MIP images of different slab thicknesses. Through the morphologies obtained from the CT slice images, the study detects small pulmonary nodules achieving a sensitivity of 94.2% [9]. Fang utilizes multi-view features of three-dimensional CT scans employing MIP for automatic detection of lung cancer nodules [32]. Drokin and fellow authors propose an end-to-end framework for detecting suspicious pulmonary nodules using MIP images based on U-Net like CNN, achieving an average sensitivity of 95% [33]. DL with MIP feature helps in achieving higher classification performance for distinguishing benign and malignant lung cancer nodules [34].

Eman et al. use histogram, thresholding, and morphological operations for lung segmentation from CT images [19]. The authors use K nearest neighbor (KNN), SVM, NB, and linear classifier for cancerous nodule classification on the TAIC dataset. The authors follow image enhancement, ROI using multi-scale amplitude-modulation frequency modulation (AM-FM), features filtering with partial least squares regression (PLSR), and training. The linear classifier performs best with 95% accuracy. Tariq et al. use a thresholding technique for lung segmentation and a neuro-fuzzy technique for the classification of the nodule with 95% accuracy [35]. Sweetlin et al. use features based on shape, texture, and run length to classify benign and malignant nodules, achieving an accuracy of 94.36% utilizing the SVM-based classification [36]. Watershed segmentation is used to segment images into different colors, making it easier to detect lung nodules in [18]. Kumar et al. present an image processing technique using thresholding and watershed segmentation for recognizing normal and abnormal nodules from nodule size. The CT image dataset had a size base of 200 mm lesions for ‘normal’ and larger than 200 mm for ‘abnormal’; hence, it can detect later-stage cancer, but this approach is not found suitable to detect early-stage lung cancer [37].

Wei Shen et al. propose multi-crop CNN, which crops nodules from CT scan cube for cancer detection. The authors use the LIDC-IDRI dataset and utilize Conv with a pooling layer to transform high-dimensional features into a low-dimensional space. Further, in the instant of the max-pooling layer, the multi-crop max-pooling layer is used for feature extraction [21]. Chon et al. propose two-dimensional deep CNN and three-dimensional CNN using 128 × 128 nodule patch and 80 × 80 × 80 nodules, respectively. Additionally, the segmented 3D RIO, linear second valine CNN, and Google Net are used. The results demonstrate that Google Net outperforms with an accuracy of 75% [22]. Ding et al. propose a fast region base convolution neural network (Fast RCNN), which extracts nodules from CT-scanned images. The deep convolution neural network is used for false positive reduction. They utilize the LUNA16 dataset and achieve a sensitivity of 94% [23]. Along the same lines, Zhu et al. propose 3D fast regions with a convolution net (F-CNN) and 3D dual-path network with a gradient boosting machine. The LUNA16 dataset is used where a 90.44% accuracy is achieved [24]. Mobiny and Nguyen use a fast capsule network for nodule classification using the General Electric and Siemens scanners dataset. With a 2D fast capsule, 89.7% precision and 87.4% recall are achieved with an error rate of 11.45 (lower than AlexNet and ResNet), while with a 3D fast capsule network, 91.9% precision and 87.4% recall are achieved with an error rate of 9.52 (lower than AlexNet and ResNet) [14].

Shakeel et al. use the cancer image archive dataset, with a weighted mean histogram equalization technique. With segmentation using improved profuse clustering technique (IPCT), deep learning instantaneously train a neural network (DITNN) and obtains 98.42% accuracy score [38]. Margarita et al. use one CNN for feature selection and one for classification. In feature selection, CNN PET and CT input images are provided to the network. An accuracy of 69.1% is achieved [39]. Gurcan and others propose multi-view light-weighted CNN for the classification of lung nodule types. In lung nodules, benign and malignant look like one point of view. However, once the point of view changes, both look different, making them distinguishable [28]. Similarly, multi-section CNN is proposed for the same task in [27]. The study uses multi-cross-sections from lung nodules for classification. LIDC-IDRI dataset is used and the accuracy of the multi-section CNN achieved is 93.18% [27].

Ali et al. propose a transferable texture CNN consisting of nine layers for feature extraction and nodules classification. Transformable texture CNN is applied to the LIDC-IDRI dataset, and the accuracy of proposed CNN is achieved to be 90.69% [28]. Veasey et al. propose a convolutional attention-based network that enables multiple-time classification in the Siamese structure, using a pre-trained 2-D convolutional feature extractor. Attention-based CNN is applied on the NLSTx dataset, and with a single time point of view, an accuracy of 85.8% is achieved, whereas, with multiple time points of view, 88.2% accuracy is achieved [29].

Clinical features such as cerebrovascular disease, diabetes, hypertension, smoking history, etc. are used with image features. 3D-ResNet is used for feature selection with the LIDC-IDRI dataset. Since LIDC-IDRI has no clinical data, this train network feature is used as an input with private hospital data and clinical data to the SVM + MKL for the nodule classification. The accuracy of ResNet-34 + MKL is 90.65% [30]. Afshar et al. propose a 3D multi-scale capsule network for lung nodule detection. The network input is a 3D nodule for local features. CapNet requires fewer data to train as compared to CNN, and the output of 3 CapNets is concatenated. A dataset of LIDC-IDRI has been used that contains 1018 samples. The accuracy of the LIDC-IDRI dataset on the capsule network is 93.12% [11].

From the literature review, the lung cancer detection CAD system can be divided into image processing-based, ML-based, DL-based and hybrid approaches. Different types of methodologies for lung cancer detection are depicted in Figure S1, given in the Supplementary Material.

## 5. Conclusions

This study involves applying various supervised learning techniques for cancer detection on the LUNA16 dataset. It envisions that the proposed model SVM + CNN, deep learning-enabled SVM outperforms all other methodologies. Understanding the dataset and extracting features from it prior to inputting data into the machine learning algorithm is necessary, as it is extremely difficult to manually select a feature from the dataset on which the algorithm can perform better. Ultimately, this feature selection impacts the final accuracy of the machine learning models. Contrarily, a deep learning algorithm also has a final classification layer, however, with a high chance of over-fitting. It also requires high computational power, a large feature set, and resources compared to machine learning models. The hybrid deep learning-enabled SVM uses the advantages of both deep and machine learning techniques. In this approach, CNN is used for feature selection and the machine learning model SVM is used for classification. No separate approach for feature selection is required in the deep learning model and raw input is provided from where features are extracted. The machine learning model cannot automatically select the features and an approach suggesting suitable features is required for classification. The advantages of both types of learning are combined in the proposed scheme and hence a definitive outcome is achieved that is more accurate and fast compared to other existing approaches.

## Figures and Tables

**Table 1:** Performance comparison of hybrid models.

Approach	Classifier	Accuracy (%)	Precision (%)	Recall (%)	F1 Score (%)
Single model	SVM	81	93	66	77
	DT	72	77	62	69
	RF	81	92	68	78
	NB	63	65	63	61
	CNN	92	93	86	89
	CapsNet	82.9	86	78	81.8
	Wide ANN	78	88	69	77.3
Hybrid model	DT + CNN	89	89	88.5	88.5
	RF + CNN	90	91.5	90	90
	NB + CNN	86	86	86	86
	SVM + CNN (Proposed)	94	95	94.5	94.5

**Table 2:** Comparison of the proposed model with state-of-the-art approaches.

Reference	Model	Year	No. of Scans	Accuracy
[18]	Watershed segmentation	2016	—	84.55%
[19]	KNN, NB, SVM	2015	166	68%, 82%, 90%
[20]	CNN Based	2018	1018	92.1%
[21]	CNN Based	2016	1018	87.14%
[22]	Google Net	2017	888	75%
[23]	DCNN	2017	1018	89%
[24]	GBM + 3D CNN	2018	888	90.44%
[5]	DCNN	2016	888	86.4%
[14]	Caps Net	2019	888	88.55%
[25]	CNN + Machine Learning	2020	100	89.14%
[26]	CNN Based, DNN-CNN Based	2017	1018	84.15%, 82.37%, 82.59%
[27]	CNN Based	2019	–	69.1%
[28]	CNN Based	2020	1018	90.69%
[29]	CNN Based	2020	15,000	85.8%
[12]	CNN Based	2019	–	85.2%
[30]	CNN + SVM	2021	1018	90.65%
Current	CNN	2022	888	92%
Proposed	SVM + CNN	2022	888	94%

## Data Availability

The dataset is publicly available at the following link: https://www.kaggle.com/datasets/yusufdede/lung-cancer-dataset.

## Supplementary Materials

The following supporting information can be downloaded at: https://www.mdpi.com/article/10.3390/cancers14215457/s1, Table S1: Acronyms used in this study. Figure S1: Types of methodologies for lung cancer detection. Figure S2: Capsule network model for lung segmentation.
