# Supplementary material for: An Effective Method for Lung Cancer Diagnosis from CT Scan Using Deep Learning-Based Support Vector Network
Source: Cancers (Basel). 2022 Nov 6;14(21):5457. doi: 10.3390/cancers14215457 (PMC9657078; doi:10.3390/cancers14215457)
Supplement: Supplementary file 1 [file cancers-14-05457-s001.zip › cancers-1922132-supplementary.pdf]

cancers

Article

An Effective Method for Lung Cancer Diagnosis from CT Scan Using Deep Learning-Based Support Vector Network

Imran Shafi, Sadia Din, Asim Khan, Isabel De La Torre Díez, Ramón del Jesús Palí Casanova, Kilian Tutusaus Pifarre and Imran Ashraf

Citation: Shafi, I.; Din, S.; Khan, A.; Díez, I.D.L.T.; Casanova, R.d.P.; Pifarre, K.T.; Ashraf, I. An Effective Method for Lung Cancer Diagnosis from CT Scan Using Deep Learning-Based Support Vector Network. *Cancers* **2022**, *14*, 5457. https://doi.org/10.3390/cancers14215457

Academic Editor: Akiteru Goto

Received: 12 September 2022
Accepted: 2 November 2022
Published: 6 November 2022

Publisher's Note: MDPI stays neutral with regard to jurisdictional claims in published maps and institutional affiliations.

Copyright: © 2022 by the authors. Licensee MDPI, Basel, Switzerland. This article is an open access article distributed under the terms and conditions of the Creative Commons Attribution (CC BY) license (https://creativecommons.org/licenses/by/4.0/).

Supplementary Material

Table S1. Acronyms used in this study.

Acronym	Full Word
AM-FM	Amplitude modulation frequency modulation
ANN	Artificial neural network
CAD	Computer-aided design
CapsNet	Capsule network
CNN	Convolutional neural network
CT	Computer tomography
DCNN	Deep convolutional neural network
DITNN	Deep learning instantaneously train a neural network
DL	Deep learning
DNA	Deoxyribonucleic acid
DIP	Digital image processing
DT	Decision tree
F-CNN	Fast regions with a convolution net
GBM	Gradient boosting machine
GPU	Graphical processing unit
IPCT	Improved profuse clustering technique
KNN	K nearest neighbor
MIP	Maximum projection intensity
ML	Machine learning
NB	Naïve Bayes
PLSR	Partial least squares regression
RBF	Radial basis function
RF	Random Forest
ROI	Region of interest
SVM	Support vector machine

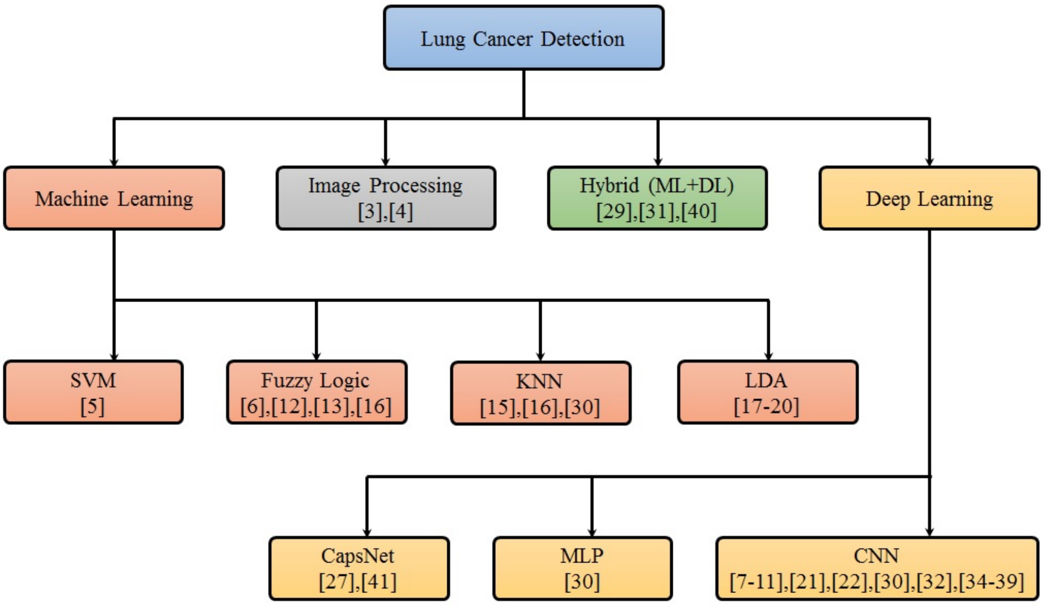

Figure S1. Types of methodologies for lung cancer detection.

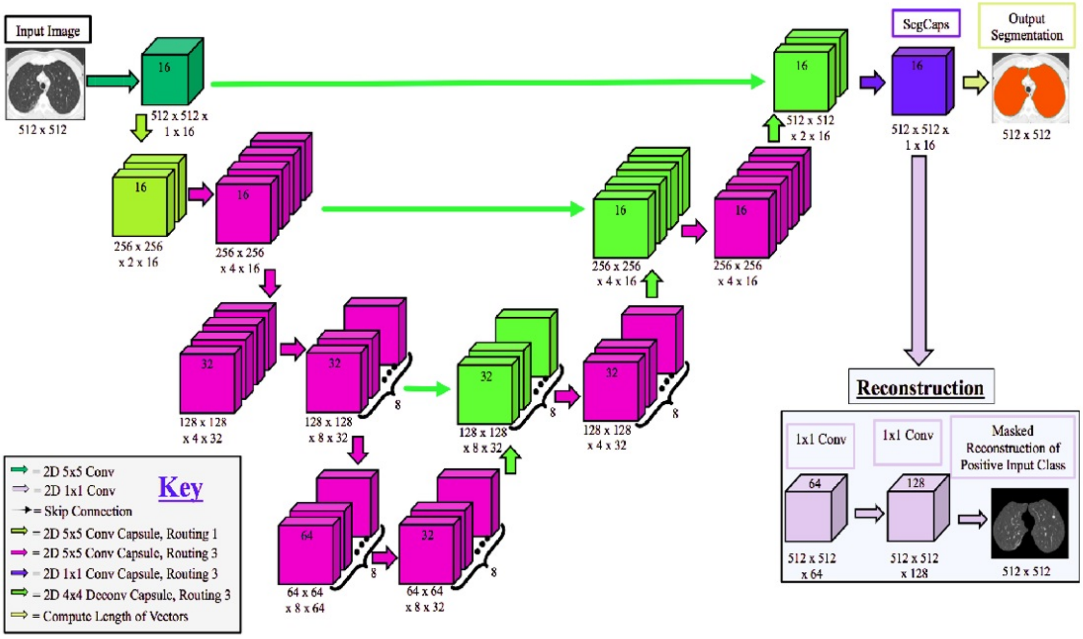

Figure S2. Capsule network model for lung segmentation.
